# Experiences of Patients With Atrial Fibrillation Using Technology to Personalize Self-Care Decision-Making: Interpretive Description Study

**DOI:** 10.2196/93036

**Published:** 2026-06-11

**Authors:** Kathy L Rush, Cherisse L Seaton, Alexandra Lukey, Victor Wang, Peter S Loewen, Wynne Chiu, Megan Patapoff, Lana Moroz, Jason G Andrade

**Affiliations:** 1 School of Nursing, Faculty of Health and Social Development, The University of British Columbia, 1147 Research Rd,Kelowna, BC, V1V 1V7, Canada, 1 250-807-8652; 2 Faculty of Pharmaceutical Sciences, The University of British Columbia, Vancouver, BC, Canada; 3 Heart Failure and Transplantation, St Paul’s Hospital, Vancouver, BC, Canada; 4 Ambulatory Cardiology, Vancouver General Hospital, Vancouver, BC, Canada; 5 Cardiac Atrial Fibrillation Clinic at Diamond Health Centre, Vancouver, BC, Canada

**Keywords:** cardiovascular, technology, self-care, experience, qualitative, naturalistic decision-making, atrial fibrillation, mobile phone

## Abstract

**Background:**

Atrial fibrillation (AF) is the most common sustained heart rhythm disorder and is a challenging chronic disease to manage. Patients’ daily self-care decisions are associated with improved AF outcomes, quality of life, and decreased hospital use and cost. However, many patients find these real-world or naturalistic decisions difficult, often because of their inherent complexity and ambiguity, coupled with the uncertainty of AF. Intervention research using technology to support AF self-care has largely emphasized making decisions with clinicians. Patients with AF are increasingly using consumer technology; yet, little is known about the use of technology by patients with AF in independent self-care decision-making. Addressing this gap will facilitate developing interventions that better leverage technology to enhance patients’ naturalistic decision-making.

**Objective:**

This study aimed to explore the experiences of older adult patients in using technology to support self-care decision-making.

**Methods:**

Following an interpretive descriptive qualitative approach, older adult patients with AF were recruited from 3 specialty heart function clinics in a Western Canadian province to participate in 1 of 6 facilitated virtual focus groups for 1.5 hours. Patients were asked about their self-care decision-making since AF diagnosis, their AF-specific technology use and its use in making self-care decisions, their technology motivations, benefits, constraints, and other possibilities for use. Inductive thematic analysis was used to code the transcribed data, moving from open coding to clustering of common codes into categories, looking for patterns of meaning between and across categories to iteratively arrive at main themes and subthemes.

**Results:**

Thirty patients (n=15, 50% women) with AF (mean age, 73, SD 5.7 years; range 63‐85 years) participated in the focus groups. Participants’ experiences of using technology to make daily self-care decisions were highly variable but centered on its personalized use to meet their individualized needs, preferences, and life context. The personalizing process of technology use in decision-making was characterized by three themes: (1) beginning technology use in their own times and ways, during their AF trajectory—pre-, at the time, at some point AF post diagnosis, and could be either self-initiated and/or provider recommended or influenced; (2) developing patterns of AF self-care decision-making using technology, including establishing their personal baseline, keeping out of the danger zone, watchful waiting, and seeking decision-making support; and (3) finding the place for technology in normalizing daily life, either settling on or limiting its use to normalize life.

**Conclusions:**

Findings expand understandings of naturalistic decision-making by elucidating the personalized process of technology use in AF self-care.

## Introduction

Atrial fibrillation (AF) is the most commonly sustained heart rhythm disorder in older adults and is a challenging chronic disease to manage. Self-care, defined as a decision-making process for managing illness and maintaining health through health-promoting practices, is a cornerstone of AF management [1]. Despite self-care’s demonstrable improvement in AF outcomes (eg, reduced complications such as stroke), quality of life, reduced health care usage and cost, and decreased treatment and relapse (eg, ablation) [2], patients with AF have found self-care decisions challenging to make [3,4]. Although providers play an important role in patients’ treatment decision-making and are a source of reassurance [5], patients spend only about 0.001% (10 hours) of their time per year with health care providers [6]. In their daily lives, patients are faced with many other decisions not directly related to treatment, such as activity, risk behaviors, and health care access [7]. Occurring in complex, real-world settings under uncertainty and ambiguity, such decisions are referred to as naturalistic decision-making.

Naturalistic decision-making has appeared in the literature since the 1980s [8]; however, its entrance into health care and cardiovascular research has been limited. Notably, research on naturalistic decision-making has demonstrated that people do not generate and compare options in systematic ways when making decisions in real-world contexts [8]; instead, they rely on past experience to efficiently categorize situations, prototypical of the Recognition-Primed Decision Model, which emphasizes situation awareness and simulating options for a course of action [9]. Similarly, with respect to cardiovascular disease self-care decisions related to medication, pain management, diet, and exercise, older adult patients did not always follow a rules-based approach in compliance with instructions from providers; instead, patients often relied on their knowledge and experience when making decisions, such as not walking when it is cold or windy, since it might affect them later on [10]. Although not specific to AF, 2 studies have explored the naturalistic decision-making processes among patients with heart failure. First, in a reanalysis of interview data, Riegel et al [11] confirmed that situation awareness (ie, recognition of a symptom and assessment of severity or importance) and mental simulation of action (ie, exploring and developing plans of action considering outcomes) were key processes underlying self-care decisions. Second, Daley et al [12] further detailed how naturalistic self-care decision-making occurred in both sequential and independent phases of monitoring, interpreting, and acting, but did not specifically study the role of technology in these processes. The use of technology in AF care has evolved rapidly and become ubiquitous, evident in the growing number of clinical trials using digital health technology for patients with AF [13]. Intervention research using digital health technologies to support self-care has primarily been clinician-driven and often embedded within integrated management systems for remote monitoring of patients’ biometric data [14,15]. For example, in a recent systematic review of AF self-care interventions, mobile and web-based interventions combined with strategies such as personalized education and continuous support showed promise in enhancing patient-reported clinical and health care utilization outcomes [15]. Despite a handful of intervention studies illustrating through qualitative follow-up methods how technology helped patients in their self-management of AF symptoms, lifestyle, and anxiety [16-19], these digital interventions were dependent on health providers, whose support, while important, may reinforce patients’ dependence on their providers for their self-care.

Independent of the health care service context, consumer technologies for heart monitoring and tracking are gaining popularity among patients with AF. In a survey administered through StopAF.org, 71% of respondents reported using consumer technology to manage their AF, most commonly KardiaMobile devices and Apple smartwatches [20]. A recent qualitative study of patients with AF using self-tracking consumer technologies reported that patients used devices to provide confirmation and reassurance of their bodily experiences and facilitate greater readiness for shared decision-making with providers [21]; however, naturalistic decision-making was not the focus of the study and little is known about how patients with AF use technology to make daily self-care decisions independently of their health care providers. Self-care is highly disease-specific [22], and naturalistic decision-making among patients with AF has been understudied. Therefore, the purpose of this study was to explore the experiences of older adult patients with AF in using technology to support self-care decision-making.

## Methods

### Study Design

This study used an interpretive description design [23], which is aligned with a constructivist and naturalistic orientation to inquiry. It is appropriate for exploratory, yet clinically applicable, phenomena such as patients’ self-care decision-making experiences using technology and the contexts in which it occurs. Reporting follows the COREQ (Consolidated Criteria for Reporting Qualitative Research) [24].

### Ethical Considerations

The study was approved by the Behavioral Research Ethics Board at The University of British Columbia (#H24-02365). All participants provided informed consent. Study data were deidentified and participant numbers were used to replace participant names so that no individual participants could be identified. Participants were offered a CAD $50 (CAD $50=approximately US $36, at the time of the study) eGift certificate for participation.

### Sampling, Inclusion, and Recruitment

Participants were recruited from 3 outpatient cardiac clinics in a Western Canadian province in the 2 months prior to focus groups using in-person recruitment, email, or telephone calls. Patient eligibility included having AF, aged 60 years or older following the World Health Organization definition of older adults [25], able to provide informed consent, able to understand English, and able to join one of the scheduled 90-minute focus groups by Zoom (Zoom Communications, Inc) or telephone. Technology use was defined broadly to ensure inclusivity of all possible sources patients might use in their self-care decision-making, and patients were given as an example “use of the internet” for this study’s purpose. Patients with self-reported limited technology use were not excluded but encouraged to join. Patients were screened from daily patient clinic lists and eligible patients either met with a research assistant in clinic prior to an appointment or were telephoned. Participants were given a high-level overview of the research and more information about the study from the consent form.

### Data Collection

Focus groups, held in June and July 2025 using the Zoom videoconference software (with telephone option), were used to elicit participants’ experiences and insights about their technology use in self-care decision-making. The virtual approach facilitated patient participation by avoiding travel logistics and costs. Before the focus groups, all participants provided consent and completed a short online survey (paper versions were offered), including demographic, cardiovascular disease, and technology-related questions. To give facilitators a sense of the technology use and confidence of participants attending the sessions, participants were asked to self-select whether they considered themselves a high or low technology user, and to rate their confidence in using technologies on a single item 3-point scale ranging from *not very confident* (1) to *very confident* (3) [26]. Participants were asked to select which of 3 devices they used (computer, smartphone, and tablet) in general and also select from a researcher-generated list of 8 digital technologies specific to cardiovascular disease management or indicate “I don’t use any.”

Focus groups, which were approximately 1.5 hours in length, were guided by a semistructured question guide asking about participants’ experiences with technologies and their use in day-to-day health and self-care decisions, with prompts used to encourage elaboration or clarification as needed (Textbox 1). Questions were broadly guided by the conceptualization by Riegel et al [11] of self-care as a naturalistic decision-making process in self-maintenance, monitoring, and managing, while integrating the role of technology. Two patient partner (1 male and 1 female) team members reviewed the proposed questions and provided feedback to simplify language. Focus groups were zoom audio-recorded and facilitated by experienced team members (KLR [PhD and female professor] and a male master’s prepared non–team member working as a research coordinator for KLR on other aging-related projects who supported data collection), both of whom had no prior relationship with participants. Other team members provided technical support and/or took notes during the session. The focus groups were fairly evenly distributed in terms of demographic and technology use composition. “Code” saturation, defined as the point when no additional codes are identified in the data, was reached at focus group 3 and “meaning” saturation, defined as the point when no further insights or nuances within the codes are identified, was reached at focus group 6 [27].

Textbox 1. **Focus group guiding questions and prompts.**

Self-care decision-making and the role of technology

1. “Reflecting on when you first received a diagnosis to present day, how have you made everyday decisions (meaning decisions that you have to make everyday about your condition) to manage your cardiovascular health and care?”
2. “How you are currently using digital technologies, meaning any type of modern device or technology, to manage your cardiovascular health and care. This includes things like smartwatches, medication reminders, online lab results, apps, or even just using the Internet to find health information. Can you tell us about any ways you are using digital technologies?”
  Possible follow-up probes (if not discussed)
  - “Specifically, can you first tell us about any ways you are using digital technologies to help you maintain your health (to support things like safe exercise or reducing risks)?”
  - “Secondly, how about using digital technologies for cardiovascular monitoring such as checking for changes in signs and symptoms (things like checking heart rate and rhythm, pulse, and blood pressure monitoring)?”
  - “Thirdly, can you tell us about how you are currently using digital technologies to manage any changes (e.g., determining treatment changes and decision making about when to seek care)?”
3. “How do you use information you are collecting from your technology to make decisions about your self-care? Probe: Can you talk about an incident using technology where you had to make a decision about your self-management? What do you do with all the information that you collect (e.g., are your providers interested in the information you are collecting)?”
4. “What motivates you to collect that data, and to use the technologies in the way that you are to look after your cardiovascular care (i.e., to maintain, monitor, and manage your cardiovascular care)? Probe: was it cardiovascular disease prompted, or a technology you used prior? Probe: who suggested the technologies—a provider or spouse or was it your independent decision?”
5. “Please talk about any benefits you have found with the technologies? What about barriers to accessing/using them—Are there any constraints that have maybe kept you from using technologies to the extent that you might or could?”
6. “Whether you are using technology or not can you imagine possibilities for using digital technologies for self-care in new or different ways than you currently are? In what ways have digital technologies met your needs in managing your cardiovascular care? Where could technology still be improved in terms of meeting your needs?”

Summary and conclusion

1. “Is there anything else related to digital technology use in cardiovascular disease self-management that you would like the team to know?”
2. “Do you have any final comments or questions in relation to this session today or this research project before we let you go?”

### Data Analysis

Participant responses to the initial survey were analyzed descriptively. Focus group recordings were transcribed automatically using Zoom software and accuracy checked against the recording by an undergraduate student research assistant (VW). Research team members listened to the audio recordings of the focus groups and read through the focus group transcripts to familiarize and immerse themselves in the data and engaged in ongoing reflexivity and reflection to promote rigor, sincerity, and credibility [28]. The approach of Richards and Morse [29] guided inductive thematic analysis moving from open coding to cluster common codes into categories, looking for patterns of meaning between and across categories to iteratively arrive at main themes and subthemes (Multimedia Appendix 1). Two team members (AL and KLR) analyzed the data, initially coding for units of meaning (eg, words, phrases, or paragraphs) across groups to construct an initial coding schema and following discussion analyzing for meaning across and within clusters of codes and refining and modifying to iteratively create themes and subthemes. Coding was completed using NVivo (version 12; Lumivero) [30].

## Results

### Participant Characteristics

Six focus groups were conducted with 5‐6 participants in each (total N=30), with 3 participants joining via the telephone option. The mean age of the participants was 73.4 (SD 5.7) years, with 15 (50%) identifying as women. All participants had at least 1 device, most commonly a smartphone. Two-thirds used a wearable and/or heart tracker of some kind, and half used online laboratory services (see Table 1 for full participant characteristic details).

**Table 1.** Characteristics of older adult focus group participants with atrial fibrillation.

Characteristic	All focus group participants (N=30)
Age (range 63-85) years, n (%)	
60‐69	7 (23.3)
70‐79	18 (60)
80+	5 (16.7)
Gender, n (%)	
Woman	15 (50)
Man	15 (50)
Ethnicity, n (%)	
European/European-Canadian	20 (66.7)
Middle Eastern	2 (6.7)
European+Indigenous	2 (6.7)
Hispanic/Latino	1 (3.3)
Indigenous (First Nations, Métis)	1 (3.3)
Prefer not to answer	1 (3.3)
Other (eg, “Canadian”)	3 (10)
Highest education level, n (%)	
University degree	19 (63.3)
Some college or university	8 (26.7)
Trades certificate/college diploma	2 (6.7)
Prefer not to answer	1 (3.3)
Annual household income, n (%)	
Over $100,000 (US $72,400)	9 (30)
$75,000 to $99,999 (US $54,300 to US $72,399)	4 (13.3)
$50,000 to $74,999 (US $36,200 to US $54,299)	2 (6.7)
$25,000 to $49,999 (US $18,100 to US $36,199)	5 (16.7)
Less than $24,999 (US $18,099)	2 (6.7)
Prefer not to answer	8 (26.7)
Marital status, n (%)	
Married/remarried	21 (70)
Divorced	4 (13.3)
Widowed	3 (10)
Single	2 (6.7)
High or low technology use (self-ranked), n (%)	
High technology user	17 (56.7)
Low technology user	12 (40)
Missing/not reported	1 (3.3)
Self-confidence in using digital technologies, n (%)	
Very confident	9 (30)
Somewhat confident	19 (63.3)
Not very confident	2 (6.7)
Device use (multiple answers), n (%)	
Smartphone	28 (93.3)
PC or Apple computer	26 (86.7)
Tablet	20 (66.7)
I don’t use any devices	0 (0)
Presence of cardiovascular disease (multiple answers), n (%)	
Atrial fibrillation/atrial flutter	30 (100)
Hypertension	9 (30)
Heart failure	4 (13.3)
Digital health technologies used in cardiovascular disease management (multiple answers), n (%)	
Online laboratory results/services	15 (50)
Online appointment booking	14 (46.7)
Wearable devices	14 (46.7)
Heart trackers/monitors	8 (26.7)
Smartphones/tablet applications	6 (20)
Online prescription refills	6 (20)
Video calls	5 (16.7)
Patient portal	1 (3.3)
Other (eg, “Holter”; “blood pressure monitor”)	4 (13.3)

During the focus group discussions, participants described their use of different technologies in AF self-care decision-making, with some avid technology users and others more limited. Common uses were technology to track biometrics, to search the internet for general health and AF-specific information, and to access health reports such as laboratory results online.

### Themes

Patient participants’ variable experiences of using technology to make self-care decisions were characterized by a personalizing process in which they tailored its use according to their unique needs, preferences, and life context. Three themes described this personalizing process across patients: (1) beginning technology use in their own times and ways, (2) developing patterns of AF self-care decision-making using technology, and (3) finding the place for technology in normalizing daily life. Each theme was defined by subthemes and participants’ perspectives, emotions, and concerns related to the process.

### Beginning Technology Use in Their Own Times and Ways

The process of personalizing technology use began for participants at different times and in different ways during their AF trajectory—pre-, at the time, and at some point of AF post diagnosis. It began with participants taking the initiative and/or because of a provider’s recommendation or influence.

#### Participant-Initiated Beginnings

An AF event often catalyzed participants’ decisions to initiate technology use in their self-care. Prediagnostically, validating troublesome symptoms (eg, feeling unwell and nearly passing out) using technology was often the deciding prompt such as 1 participant who purchased their own smartwatch immediately after using a friend’s watch that showed AF on an electrocardiogram (ECG) and confirming with their doctor (Participant 8).

An AF diagnosis was the impetus for several participants to initiate technology use, either acquiring new technology for the first time, adding AF-specific technology (eg, KardiaMobile) to what they already owned, or using their current technology for their AF self-care. AF was the sole reason for several participants to purchase technology, such as Participant 8 who admitted, “But the reason I wear it [smartwatch]- I never would have bought it if it hadn’t been for the atrial fibrillation” while Participant 26 echoed having “no need for a smartwatch prior to [AF diagnosis]” but described it as “a [game] changer for me.” Another participant described blending their current with newly acquired technology “brought on by my heart condition” (Participant 28).

#### Provider Recommendations and Influences

Multiple participants described the influence of their physician’s recommendations or perceptions of the technology (eg, tracking device) in their decision to begin and/or continue using technology. Participants described providers giving them practical help to maximize the use of their existing technology (Apple Watch to run ECG) or asking them to “run a couple of strips and email them to me if you ever have issues” (Participant 23), or to have copies of the data for their records. However, not all participants responded as positively to provider recommendations to use monitoring devices, with some expressing greater reluctance as one participant described, “I’ve been recommended to use all those things, and I’m pretty slack at it, but it’s a matter of getting used to doing it all the time, and not begrudging the fact that you have to” (Participant 16).

In contrast, a number of participants described providers who were less receptive and interested in their technology data, which influenced their decision-making. Lack of provider interest led 1 participant to decide against paying the additional costs to upgrade and connect their KardiaMobile to the internet to transmit data (Participant 9). One participant, who used only a step counter app for measuring walking steps and no other device for AF monitoring noted that “my doctor hasn’t really suggested any devices so far” (Participant 20).

Even when doctors recommended technology use, less technologically savvy patients were frustrated by their lack of digital literacy either not using it because they don’t “know how to work the thing” (eg, KardiaMobile) (Participant 4) or delaying device adoption until their AF became uncontrolled, as one participant elaborated,

*My GP [general practitioner] and my cardiologist wanted me to have an Apple Watch. And I thought, oh great, just another piece of technology that I will not understand. So eventually my AFib got out of control, and so I marched myself over to the Apple store.*

[Participant 25]

### Developing Patterns of AF Self-Care Decision-Making Using Technology

#### Overview

Regardless of when participants initiated their use of technology, they described using 1 or more devices singly or in combination for making daily self-care decisions. Participants personalized their use of technology in their AF self-care decision-making based on several contextual factors: type of AF (symptomatic or asymptomatic), daily activity, technology preferences, comfort levels, data relevance to their situation, and whether responding to specific AF episodes or proactively to mitigate AF exacerbations and their consequences. Overall, participants were sophisticated active data managers and processors evident in their use of 1 or more of 4 technology-driven decision-making patterns: (1) establishing their personal baseline for decision-making, (2) keeping out of the danger zone, (3) watchful waiting, and (4) seeking decision-making support. The ability to “self-diagnose” and confirm episodes of AF through technology was lauded as a way of gaining some control, when in the past participants had to wait for information from their providers, *“*It’s really great to have any kind of diagnostic tool, just to have a feeling of empowerment and learning more about your own condition, instead of being in the dark as way back in the past*”* (Participant 12).

#### Establishing Their Personal Baseline for Decision-Making

Participants found that technology facilitated learning more about themselves in the context of their AF to personalize their self-care decision-making. Despite variability in the consistency, frequency, type, amount, and sources of data they gathered, core to participants’ self-care decision-making was establishing their baseline. Participants consistently referred to using their data to “benchmark,” “trend,” “pattern,” and “compare” that was foundational to determining changes in their condition that informed daily decision-making.

Several participants did very extensive tracking, trending, and analysis of their AF data over time, using their preferred method of either paper or devices/apps. Some participants had been doing this for several years—one since their cardiologist’s recommendation 20 years ago and “seeing if blood pressure is going up or is too low” to make decisions (health care seeking) (Participant 13) and another having saved their laboratory results for the last 10 years “to compare them [and] if off kilter, say[ing] okay, we’ll figure out why” (Participant 1). Participants tracked their own trends to understand, inform, and personalize their discussions with providers, as one participant elaborated of using an Excel spreadsheet, to “track everything” noting when they experienced spikes, and graphed trends over time to “be able to join the picture and get right in it and discuss it” with their cardiologist (Participant 23).

In establishing their baseline, participants considered data accuracy as important, at times expressing distrust or concerns about the reliability of their devices and the implications for their decision-making. One participant with a smartwatch questioned the reliability of their smartwatch when it “reported different numbers” compared with that of hospital heart monitors (Participant 30). In deciding about the need for an emergency department (ED) visit “when it [AF] just didn’t stop,” another participant, who had a smartwatch took “my pulse just to make sure the watch was working” (Participant 3). Between 2 devices’ (KardiaMobile and FitBit Sense) reading discrepancies, another participant preferred to rely on their Fitbit as, “my condition looks worse on the Kardia[Mobile] than it does on the Fitbit” (Participant 18). Others had done research into the accuracy of different devices for AF to decide which to use for establishing their own personal baseline. Yet, another participant described the information from their digital technology that varied daily as establishing their own personal reference points:

*It’s like standing on a scale when you go to the doctor’s office—it tells you you’re always something different than you are at home. But it’s relative. I was this much today, and I’m that much tomorrow. So it’s all relative to your own personal information.*

[Participant 18]

#### Keeping Out of the Danger Zone

Keeping out of the danger zone was a proactive approach in which patients used technology to “self-diagnose” and validate AF episodes both for making decisions in the short and long terms. Short-term decisions were made to avoid personal risks of triggering an AF episode or respond to symptoms in the moment while long-term decisions focused on preventing complications such as stroke. For example, participants discussed using tracking technology together with their symptoms to monitor for AF triggers to inform behavior change. One participant shared, “I found that my Apple Watch confirmed what my symptoms were already telling me...if it is happening more, I need to change my behaviors i.e. alcohol intake, caffeine intake, amount of social involvement/volunteer commitments, and personal stress” (Participant 19). This was echoed by another participant who explained that in the short term, they could react and change behaviors in the moment, but also, “I think, in more the longer term the technology gets me better informed what I should or shouldn’t be doing” (Participant 27).

Participants’ use of technology to keep out of the danger zone was similar whether their AF was symptomatic or asymptomatic. Asymptomatic participants found ongoing tracking invaluable to alert them to AF episodes of which they would otherwise be unaware. Several participants found continuous wearable tracking devices highly valuable, such as “detecting AF during the night when you’re asleep” (Participant 14). Even symptomatic participants used continuous wearable technology such as one participant who had experienced a missed AF on a Holter monitor that a continuous wearable detected and was accepted by their provider. Both symptomatic and asymptomatic participants used technology to reduce fear and uncertainty related to their AF risks. For example, technology use reduced an asymptomatic participant’s fear of a stroke, “Because with atrial fibrillation, you don’t always know when you don’t always feel it. I live in fear of having a stroke, and so it just gives me peace of mind” (Participant 4). Symptomatic participants similarly used technology to allay uncertainty, such as one participant with AF symptoms that were “not subtle,” who wore a smartwatch daily as a “backup” to allow them to confirm “yeah, this is happening right now, and it started happening then” (Participant 17). One participant who was not considering using a smartwatch until participating in the focus group (despite her brother’s recommendation) entertained the value of being able to confirm AF episodes, “You know, because sometimes I think, oh, I wonder if that’s AFib, or if it’s just because I walked up the big hill” (Participant 20).

Other participants personalized when and what technology they used to meet their individual needs, such as when symptomatic episodes arose, or whether asymptomatic for intermittent monitoring, or just during certain activities (such as exercising). One asymptomatic participant, who never knew when he had an elevated heart rate (“up to 200 or 150”) periodically checked his biometrics, with blood pressure or heart rate monitor and KardiaMobile as “without that, I often wouldn’t even know I’m having a problem” (Participant 9). Another participant who used a chest strap heart monitor only when exercising also had a KardiaMobile, blood pressure monitor, and oximeter, and limited using these only if symptomatic, admitting, “Most of the time it’s through a self-assessment of how I feel during the day and night...If I don’t have those symptoms, I don’t do anything” (Participant 28). Three participants described using a heart rate strap with their smartwatch to accurately monitor their heart rate response to exercise and use the data to pace themselves to avoid “stress on my heart” (Participant 28), as one participant described:

*If I’m out for a long run, and I suddenly find that my heart rate, because of the strap, has me over 140 or 150, which isn’t that unusual, what I’ll do, only because I don’t want to red line things, I’ll just back off. And I’ll slow down, so it allows me to manage my exercise routines as well as my heart health care if it gets into sort of high-risk situations.*

[Participant 27]

Other participants used technology for their personal medication management. Participants described using technology to promote medication compliance, such as setting a smartwatch “alarm to remind [me] to take [my] medication” (Participant 18). One participant recalled a specific incident of how technology provided data that allayed their risk-related concerns and gave reassurance as to the effectiveness of their medication management self-care:

*When my resting heart rate was 104, I was quite concerned about that high level of resting heart rate, but subsequently that has gradually gone down to 78 now. So that helps me detect, you know that I’m taking the right medications and know the medications are working. So that’s my feedback mechanism to help me realize that, you know, I’m not in the danger zone.*

[Participant 30]

#### Watchful Waiting

Watchful waiting was a prevalent approach participants used specifically during episodes of AF in making decisions whether or not to seek health care. Watching and waiting was a very active decision-making approach in which participants engaged in 1 or more of the following over time that varied from a few hours to days: watching their digital device and biometric monitor data closely, observing changes in values relative to normal range over time, listening to their symptoms (if they were symptomatic), considering their larger context (eg, what they were doing and what might be triggering), and putting the data together over time to decide whether to seek care. A number of participants were exuberant about the lifesaving role of their tracker that was reinforced by some of their providers.

Technology made a significant difference for participants in their decision-making about seeking ED care. Several participants described watching and waiting using their technology data only, and/or coupled with various sources of data, they processed to inform their decisions. Some sought ED care earlier, later, or not at all, the time frame often varying according to the types and number of data sources they used. Those who sought emergency care earlier often relied exclusively on their immediate device readings, such as one participant who went to the ED within 2 hours of KardiaMobile confirming AF “after one hour of it going on” but which had stopped by ED arrival (Participant 12). Using data from 2 devices (smartwatch and heart rate monitor) to confirm prolonged AF (4 hours), together with considering their activity and symptoms (vague or very mild), another participant decided “pretty quickly that I’ve got to head into a clinic, or somebody’s got to see me pretty fast, and you’re off to the emergency room” (Participant 27).

In contrast were some participants who watched and waited over several days and varying activities, during which they monitored their subjective symptoms and objective device data before deciding to seek care. For example, one participant used night-time alerts during sleep showing a high heart rate (180) and a few days later experienced difficulty keeping up during activity that prompted a doctor visit, “I went for a little bike riding [with grand-daughter] and I was having trouble keeping up with her. And I’m out of breath. Something’s wrong here, plus the alert. So I went to the doctor” (Participant 18).

Some participants described avoiding unnecessary ED visits because of the objective reassurance their tracker data gave them despite subjective symptom severity,

*I would sort of look at it [smartwatch], and my symptoms are really bad, and I’d watch, and I’d think, ‘Okay, well, I’m going to give this another few hours.’ But I never went to emerge [ED] ever because the watch reassured me that, you know, I was okay hahaha. So I never got frightened that way. I think if I didn’t have the watch, I might have been more nervous. I might have been maybe seeking more emergency care*.

[Participant 19]

#### Seeking Decision-Making Support

Despite settling into patterns of independent daily decision-making, participants recognized the need to supplement these in some situations related to their AF self-care often looking to others for support. However, there was considerable variation in participants’ need for support that reflected either their comfort or cautiousness in using the data collected from their different technologies—tracking devices, laboratory work portal, and internet—for self-care decision-making. One participant captured this variability, contrasting his wife’s “insisten[ce] looking at her results as soon as they come up [and] panics over every number that’s outside the normal range, [while] I refuse to go and look at my results. I wait for the doctor” (Participant 3).

Participants expressed considerable awareness of the limitations of relying solely on their technology data for self-care decisions and described situations warranting deferral to their providers. Data misinterpretation was their chief concern whether from lack of context, knowledge, or potential for “life and death” consequences. Participants regularly used online information sources including medical websites (Mayo Clinic) and ChatGPT (Participant 13) for gaining knowledge or answers to their questions about their personal health situation, such as implications of comorbidities (autoimmune diseases) for their AF (Participant 11). Yet, they exercised caution and wanted to check it out with their physicians despite some experiencing decisional pressure from “a lot of people, friends, and relatives—Oh do this, do that, I have done that” (Participant 13). Understanding and interpreting laboratory results were highly variable with some participants comfortable because of previous background (eg, medical field) and others uncomfortable. Participant 4 expressed their discomfort with interpreting laboratory results, comparing the difference between using “trial-and-error” for YouTube music and “dealing with your personal circumstance” but continued tracking patterns and trends after discussing with their general practitioner, blending provider support with independent self-care decision-making. Other areas for which participants wanted supplemental information related both to optimizing their technology data interpretation, such as “all the information I get from my [smart]watch (e.g., nighttime bradycardia)” (Participant 2), and translating their online searches into personal application for their decision-making. One participant, who wanted help with the difficult decision of “when it’s time to go to emerge [ED]” wanted more help with data interpretation and suggested use of “Artificial Intelligence, that ‘would [be] require[d] to do the work of doctors’” (Participant 17).

### Finding the Place for Technology in Normalizing Daily Life

Participants’ desire to normalize life with AF was a consistent refrain in their accounts, but the role of technology in doing so varied. For many, it facilitated their feeling of normalcy, while for others it detracted.

#### Normalizing Life With Technology Use

Several participants found that technology use helped normalize life. Some found that technology gave peace of mind and reassurance, allowing them to get on with life. According to one participant, “I love technology. I almost want to use it so I can get instant information, put it aside, and get on with my life” (Participant 23). Device ECGs showing that they were in normal sinus rhythm were highly reassuring for participants and contributed significantly to normalizing their lives. One participant experienced this reassurance through the habitual use of technology to take smartwatch readings every morning,

*Most mornings I wake up, and I feel like I’m in AFib. So it’s almost more of a method of sort of proving to myself that I’m not in AFib. So I’ll just take a reading as reassurance “No, you’re fine get on with the day.”*

[Participant 26]

Technology and specifically the use of home heart rate or rhythm tracking facilitated participants’ decision-making confidence in responding to their AF symptoms that helped normalize life and reduce anxiety. Some also talked about needing to track less as they became more attuned to their symptoms, as one participant elaborated:

*I only use it if I sense it goes wrong. I’ve had, over the years, I really get this, I can really sense my heart. I know if it’s starting to feel like a bouncing tennis ball, or if it starts to do something, I’ll get the KardiaMobile out, and then I’ll just run a, you know, a few strips.*

[Participant 23]

#### Normalizing Life With Limited Technology Use

Limiting technology use helped normalize life for other participants. They decided that *not* dwelling on AF through the overuse of technology contributed to their sense of normality and reduced stress. Participant 20, who initially expressed discomfort using a personal device because, “if you’re obsessing over your AFib, you’re stressing yourself out” which she considered a possible AF trigger, normalized life by just sticking with her doctor’s prescribed testing or monitoring. Reinforcing this sentiment, another participant agreed, saying “don’t fuss over AFib. Just [live] life as normal as possible” (Participant 3). Another participant who admitted that they could *“*get to be a bit obsessive about data...but did not have a context in which to analyze it” normalized life by letting go of the data obsession and now monitors only their blood pressure periodically at the recommendation of their general practitioner, and “I just look at spikes, otherwise I just get on with life” (Participant 4). A participant who had “faithfully” monitored blood pressure for 2 years after diagnosis until the COVID-19 pandemic and had not resumed, “I’ve just kind of, I know when I’m in AF, I can feel it and I change what I do, kind of calm myself down a bit, and it seems to help it stop,” is no longer monitoring except occasionally checks it out as symptoms arise, “otherwise I’m just living my life the way I used to. Not worrying about it” (Participant 5).

Despite settling into a rhythm with technology as a tool to help them get on with their lives, for some there was an underlying uncertainty about how technology could render them AF-free which for them would return them to a sense of normalcy. Ultimately, one pondered the process of coming to the place where technology fits in self-care decision-making, amid the uncertainty of their AF trajectory,

*I find all of this is allowing me to monitor, and I guess, manage, on a day-to-day basis. But in terms of what it means in the greater picture, I really don’t know. That to me is kind of the ultimate end. Some sort of resolution to this*.

[Participant 27]

## Discussion

### Principal Findings

This study provides novel qualitative insights into the experiences of older adults with AF using digital technology to make decisions about their daily self-care. The extent to which patients made use of technology in their self-care decision-making was highly personalized as they considered their context, symptoms, and activities. This process was characterized by several interrelated themes, beginning with either patient-initiated or provider-recommended use, and developing into patterns of digitally supported decision-making, and finally finding the place for technology within their life context.

Findings reflected that technology facilitated participants’ independent or naturalistic decision-making about their daily AF self-care, which is largely performed by patients outside of health care settings [6]. Similar to another study of patients with AF, whose use of heart-tracking technologies gave confirmation and reassurance about their bodily symptoms, patients in this study often gained self-care decision-making confidence and reassurance from their technology-collected objective data [21]. This resonates with and extends the naturalistic decision-making literature, including the illustration of the Recognition-Primed Decision Model in self-care decision-making by Riegel et al [11] and the process by Daley et al [12] regarding monitoring, interpreting, and acting among patients with heart failure by showcasing the growing centrality of technology in decision-making for both habitual and irregular users. AF’s unpredictability and uncertain course make it a highly challenging condition to manage, often leaving patients feeling left to trial and error [3,4]. Our in-depth look at patterns of daily self-care decision-making highlighted ways older adults with AF used technology to improve their situation awareness (eg, reduce symptom uncertainty or ambiguity), supporting sophisticated naturalistic decision-making.

Our participants recognized the value of continuous monitoring with wearable devices and smartwatches to make actionable decisions to avoid triggers, reduce risks, modify lifestyle behaviors and daily activities, and guide health care seeking. These findings contrast with those from a study on technology use among patients with rheumatoid arthritis, who questioned the value of pain monitoring and tracking if it was not going to lead to any change in their management [31]. This divergence suggests that self-care decision-making using technology is highly disease-specific [22]. A comparative qualitative study of the self-care decision-making between patients with type 2 diabetes, HIV/AIDS, and multiple sclerosis found that there were common yet disease-specific self-care decisions participants made because each disease has unique attributes that influence the meaning and interpretation of specific self-care decisions [32].

Participants in this study made judicious use of health care services and tried to avoid unnecessary ED visits, as they used watchful waiting strategies in making their decisions. Although concerns have been raised about the potential for consumer device use to prompt greater health care use in patients with AF [33,34], our participants detailed decision-making experiences of knowing *better* when to seek health care, reinforcing notions that technology holds great potential for win-win solutions that both support patients and lessen burden on health systems [35]. Accounts from individuals who used self-tracking technology pre–cardiac diagnosis also resonate with our findings as they collected data over time, compared with their norm, and continued to monitor before deciding to seek care that ultimately led to diagnoses [36]. Nevertheless, the use of consumer devices for widespread AF screening is highly contested and carries potential for medical overuse among undiagnosed people who are unlikely to gain clinical benefit [37].

Although participants in this study emphasized their independent use of technology for making their self-care decisions, they also collected, tracked, and trended their data to share and/or discuss with their providers and deferred to them to meet their need for support interpreting data (eg, laboratory values) in making decisions. In previous intervention research, participants who provided a smartphone app for AF management expressed disappointment to find that there was no mechanism for the data collected to be shared directly with their providers [7]. Although our participants were not directly provided devices, they too were often seeking decision-making supports to process their personal data. Several patients in this study also expressed concerns about anxiety and becoming overly preoccupied with tracking. Indeed, the potential for consumer heart-tracking devices to provoke anxiety in patients has been highlighted in other work [38]. In an intervention study of 14 patients with AF who were provided several monitoring devices, many found the measurements motivational, but 2 patients expressed concern about overmonitoring [19].

Findings revealed variability in the way patients normalized their use of technology for self-care decision-making. For many patients, technology supported informed decision-making that provided them reassurance, allowing for a return to a sense of normalcy, whereas for others, normalizing life was not becoming preoccupied with the use of technology that kept them fixated on their AF. Similar to the process of finding normality in living with chronic disease [39], patients integrated technology within that process as they adapted and constantly defined and redefined normalcy. Overall, technology use for achieving normalcy had to align with patients’ personal preferences, reflected in the growing body of literature on the need for technology to be personalized according to the patient and the purpose [40,41]. Importantly, consideration must be given to contextual factors (eg, cost, preferences, and digital literacy) that influence technology use to avoid reinforcing unequal access to health care services and disparities in socioeconomic status for marginalized older adults with AF [42].

These findings have implications for providers, patients, researchers, and policymakers. While a care goal of providers is to encourage patient self-care and active involvement in their care, they are often limited in supporting patients’ naturalistic decision-making, for example, in implementing and sustaining lifestyle changes [43]. Given this study’s elucidation of perceptions of patients with AF regarding the value of technology to support their self-care decision-making, providers might choose to encourage the use of these tools. Supporting them, however, may require additional provider education and training [42]. The 4 decision-making approaches observed in this work hold potential to inform the development of patient-facing tools to better support patients with AF in naturalistic decision-making by building their capacity to interpret and process the growing volumes of data they obtain with technology and to reduce clinician burden. Canadian Cardiovascular Guidelines for AF [44] include recommendations related to self-care and lifestyle but have not yet incorporated the role of consumer devices in these recommendations. Policy guidance is needed on best practices to inform patients and providers on the most appropriate technologies, personalizing use to patient needs, and a balanced incorporation of consumer data in decision-making.

### Limitations

Focus group participants were well educated, were fairly active users of technology in general, and had higher incomes, which may limit the generalizability of our findings to others. The cost of consumer devices, for example, may be a significant barrier in other samples that include greater representation of patients with lower socioeconomic backgrounds. Virtual focus groups allowed participants to join from wherever it was most convenient for them and may have attracted a biased sample, although 3 participants did join using the telephone option. Although we reached participants with a broad range of technology use, including those who described themselves as low technology users, the sample may not adequately represent marginalized or genuinely low-technology populations, given all participants used at least 1 device (computer, smartphone, or tablet), and technology use was defined broadly (eg, “use of the internet”) during recruitment. Nevertheless, the proportion using heart trackers mirrored that of a national survey [20].

### Conclusions

This study contributes the first in-depth qualitative insights into the use of technology in naturalistic decision-making among older adults with AF. Our patient participants used technology to personalize everyday AF self-care, outlining intricate decision-making processes. These findings have implications for supporting decision-making tailored to individual needs and context while considering the broad range of technology use among patients with AF. The findings can also form the basis for continuing work in this area to further investigate the role technology plays in patient decision-making and quality of life, as well as health care use, costs, and provider burden.

## Supplementary material

10.2196/93036 Multimedia Appendix 1 Illustration of main themes derived inductively through initial codes and subcategories identified.

## Abbreviations

AF: atrial fibrillation
COREQ: Consolidated Criteria for Reporting Qualitative Research
ECG: electrocardiogram
ED: emergency department
