# Supplementary material for: Experiences of Patients With Atrial Fibrillation Using Technology to Personalize Self-Care Decision-Making: Interpretive Description Study
Source: JMIR Cardio. 2026 Jun 11;10:e93036. doi: 10.2196/93036 (PMC13256484; doi:10.2196/93036)
Supplement: Multimedia Appendix 1 [file cardio-v10-e93036-s001.docx]

Multimedia Appendix 1

Table S1. Illustration of main themes derived inductively through initial codes and subcategories identified.

| **Initial inductive codes** | **Emerging sub-themes** | **Final sub-themes** | **Final themes** |
| --- | --- | --- | --- |
| Personalizing technology choices | Participant initiated technology use | Participant initiated beginnings | Beginning technology use in their own times and ways |
| Turning points in catalyzing individual technology use |  |  |  |
| Receiving provider guidance in technology use | Provider influences on technology use | Provider recommendations and influences |  |
| Positive or negative provider technology receptivity |  |  |  |
| Encountering barriers (e.g., digital literacy) | Barriers to adopting provider prescriptions |  |  |
| Tracking and recording Information/data (e.g., analog vs digital) | Gathering personal biometrics | Establishing their personal baseline for decision-making | Developing patterns of AF self-care decision-making using technology |
| Learning their normal AF metrics through technology |  |  |  |
| Trending data over time | Processing and applying data |  |  |
| Examining device accuracy |  |  |  |
| Avoiding short- and long-term risks | Reducing high heart rate risk | Keeping out of the danger zone |  |
| Reducing AF triggers |  |  |  |
| Managing medications | Reducing complications (e.g., stroke) |  |  |
| Time variation in seeking care (deferral) | Watching and waiting and waiting and seeing | Watchful Waiting |  |
| Deciding to go or not to go to ED |  |  |  |
| Making sense of signs and symptoms | Integrating data sources |  |  |
| Recognizing limitations in deciphering data | Supplementing data interpretation with other sources | Seeking Decision-Making Support |  |
| Needing support |  |  |  |
| Feeling reassured with technology | Leveraging technology in daily life | Normalizing life with technology use | Finding the place for technology in normalizing daily life |
| Gaining confidence with using technology |  |  |  |
| Obsessing over tracking data | Finding technology counterproductive | Normalizing life with limited technology use |  |
| Finding technology anxiety producing |  |  |  |
